# The Mechanobiology of the Actin Cytoskeleton in Stem Cells during Differentiation and Interaction with Biomaterials

**DOI:** 10.1155/2018/2891957

**Published:** 2018-10-08

**Authors:** X. Ambriz, P. de Lanerolle, J. R. Ambrosio

**Affiliations:** ^1^Departamento de Microbiologia y Parasitologia, Facultad de Medicina, UNAM, 04510 Ciudad de México, Mexico; ^2^Department of Physiology and Biophysics, College of Medicine, University of Illinois at Chicago, 60612 Chicago, Illinois, USA

## Abstract

An understanding of the cytoskeleton's importance in stem cells is essential for their manipulation and further clinical application. The cytoskeleton is crucial in stem cell biology and depends on physical and chemicals signals to define its structure. Additionally, cell culture conditions will be important in the proper maintenance of stemness, lineage commitment, and differentiation. This review focuses on the following areas: the role of the actin cytoskeleton of stem cells during differentiation, the significance of cellular morphology, signaling pathways involved in cytoskeletal rearrangement in stem cells, and the mechanobiology and mechanotransduction processes implicated in the interactions of stem cells with different surfaces of biomaterials, such as nanotopography, which is a physical cue influencing the differentiation of stem cells. Also, cancer stem cells are included since it is necessary to understand the role of their mechanical properties to develop new strategies to treat cancer. In this context, to study the stem cells requires integrated disciplines, including molecular and cellular biology, chemistry, physics, and immunology, as well as mechanobiology. Finally, since one of the purposes of studying stem cells is for their application in regenerative medicine, the deepest understanding is necessary in order to establish safety protocols and effective cell-based therapies.

## 1. Introduction

**Figure 1:**
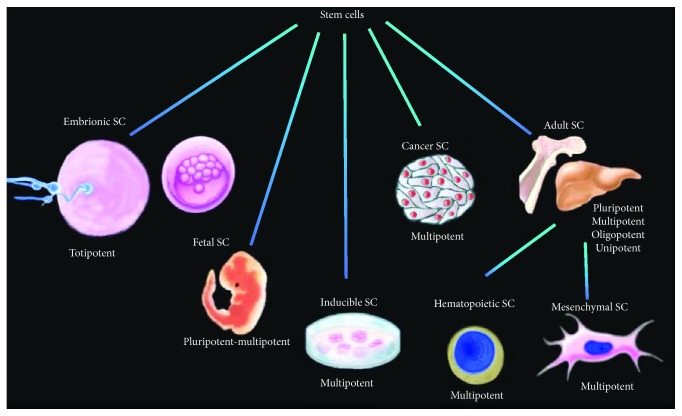
Kinds of stem cells and their differentiation potencies. Stem cells can be obtained from various tissues, with different potential properties (by Dr. Ambriz, 2018).

Stem cells are undifferentiated cells with the potential to generate diverse lineages, but they are also capable of maintaining their own population, a process well known as self-renewal. Stem cells can be obtained from various tissues, with diverse potential properties, being able to generate from one to all kinds of cells (Figure 1).

Embryonic stem cells (ESCs) are isolated from the blastocyst and have the potential to generate any kind of cells from the three germ lines: ectoderm, mesoderm, and endoderm [1]. Mouse ESCs have been intensely studied for their capability of self-renewal, totipotency, and genome stability in comparison to human ESCs [2]. The interest in these kinds of cells is not solely for totipotency and regenerative use, but also for immunotherapy as well as a vehicle for drug delivery. At the moment, the use of ESCs in cellular therapy is controversial, due to ethical issues requiring human oocytes in obtaining these cells. Despite their legal use in some countries, most other countries prohibit the use of this tissue.

Inducible pluripotent stem cells (iPS or iPSCs) are generated by viral transfection of fibroblasts from adult humans, with these key transcriptional factors: Oct4/3 (octamer-binding transcription factor 4/3), Sox2 (sex determining region Y), Klf4 (kruppel-like factor 4), and c-Myc (avian myelocytomatosis virus oncogene cellular homologue) [3]. This strategy generates “stem cell-like” cells similar to the ESCs. They both share ethical controversy, but in this case, because iPSs are generated by viral transfection and because the stability of the incorporated genes is still unknown, this issue has to be solved before using iPS in humans.

Adult stem cells or somatic stem cells, also referred to as tissue-specific stem cells, are cells that can be obtained from already born animals and humans, not necessarily adults, because infants also have adult stem cells. These stem cells are necessary to maintain the body during its lifetime, with a self-renewing capability but without the potency to generate cells from the three germ lines.

Mesenchymal stem cells (MSCs) are a type of adult stem cell that is self-renewing and pluripotent. MSCs have the capacity to differentiate into several lineages, mainly adipocytes, chondrocytes, and osteocytes. On the other hand, hematopoietic stem cells (HSCs), another kind of adult stem cells, have the potential to generate blood cells like lymphocytes, dendritic cells, natural killer cells, monocytes, and others, while neural stem cells (NSCs) can generate lineages from the nervous system, neurons, and glia (astrocytes and oligodendrocytes).

Cancer stem cells (CSCs), also known as “cancer stem-like cells” or “tumor-initiating cells” (TICs) are a kind of stem cells which may express surface markers present on human ESCs and/or adult stem cells [4]. These cancer cells share the same properties of self-renewal and differentiation with stem cells, and for that reason are included into this category. CSCs are defined as cells capable of generating many cancer types and the failure of chemotherapy, which will be discussed later.

In order to regulate the recovery and characterization of stem cells, the International Society for Cellular Therapy (ISCT) established the minimum criteria to define them as stem cells [5], including specific recommendations that need to be followed in order to identify and avoid “unproven cellular therapies,” any manufacturing of products, and loss of trust in the field. Furthermore, the ISCT strongly encourages the sharing of efforts and the contributions of involved professionals, as well as establishing the identification of key features of unproven cellular interventions. In this context, in order to have standard culture conditions for the maintenance of stem cells and the possibility of testing the effect of any kind of biomaterial on these cells, it is mandatory to elucidate intracellular events produced by the involvement of the cytoskeleton and mechanotransduction, which is the transduction of mechanical stimulus into intracellular signaling, both chemical and biophysical. Moreover, a higher scope of knowledge of these events and description of the involved mechanism will result in an increasing confidence for new cellular therapy protocols.

Steady-state conditions and differentiation processes are key aspects for the establishment of stem cell culture. However, the limitations involved in the access to organs or prostheses narrow the study of the implantation of stem cells, pushing more attention into the development of cell therapies [6].

Additionally, other important challenges that require examination are phenotype stability and maintenance in culture conditions, self-renewal and control of lineage commitment to ensure the identity of stem cells introduced to a body and, finally, the stability of a specific phenotype inside the body, avoiding aberrant differentiation, like that of cancer-like cells.

Biophysical aspects, such as the role of the actin cytoskeleton-mediated mechanobiology of stem cells, are important to consider. The conjunction and comprehension of these aspects present a better way for establishing all the possible interactions between biomaterials, cells, and organisms in order to be used for medical purposes.

The ability to design biomaterials to mimic natural scaffolds is a novel perspective for improving or developing more efficient stem cell-based therapies in regenerative medicine, requiring a deep understanding of stem cell biology.

Considering that regenerative medicine is a promising field for developing and applying new medical therapies, based on stem cell use, there is a continuous necessity to explain how these cell types are controlling self-renewal and differentiation. In this context, we could be close to developing “living implants” for humans, with their high capability of integration to a body without rejection, as well as the induction of a cellular replacement in situations of tissue damage, using the extracellular components of the host.

## 2. The Role of Cytoskeletal Proteins in Stem Cells

The cytoskeleton is a highly dynamic web composed of different molecules including actin, tubulin, and vimentin, whose role is dependent on the context and structures generated in each condition, but it also relies on the cell type. In this way, the cells have a specialized manner to respond to the environment, always trying to survive and adapt. The response of adherent or nonadherent cells involves different mechanisms, including surface molecules, like integrins and selectins that help with adhesion, and generate a link between the physical microenvironment from outside and within the cell. Thus, the cytoskeleton provides the support for stem cells in culture conditions or homing and establishment inside the body. Considering that, in both cases, the mechanisms are not completely understood, this section focuses solely on the physical signals the cells receive, excluding soluble factors, which are also relevant, as most of the literature already addresses them.

Cytoskeletal rearrangement is induced by several stimuli like chemokines, growth factors, differences in the stiffness of a substrate, and others. As mentioned above, the extracellular environment is connected to the inner cell, including the nucleus [7, 8]. In that case, the characteristics of the substrate may generate a reaction inside the cells, and the reaction is led by a reorganization of the F-actin structuring and the modulation of actin dynamics, like polymerization/depolymerization cycles.

In this context, actin reorganization is required during stem cell differentiation, as well as adhesion, cellular spreading, force distribution, stress fiber formation, and others, all of which are completely dependent on the cytoskeleton, a complex scaffold constituted of different kinds of filaments distributed throughout the cytoplasm. Thus, rigidity, structure, and support are not the only functions, but also subcellular organization, inner transport of molecules, motility and migration, cellular division, and mechanotransduction [9–11].

## 3. Cytoskeletal Structures Implied in Cellular Morphology and Lineage Commitment

Stem cells have a fibroblastic shape, which is considered as one of its morphological characteristics. There are specific structures that may serve in the stem cell progression of differentiation. These include the cytoskeleton and specific structures generated to make possible to establish stem cells in culture, as well as in stem cells during differentiation to specific lineages [12–17].

Osteogenic differentiation, induced by soluble factors, showed modification of the actin cytoskeleton. This morphology modification is a consequence of parallel arrangement and orientation of the actin filaments, to the rearrangement of F-actin in well-defined stress fibers as well as a change in its pattern. FA distribution and cellular density are altered by physical characteristics of the substrate and/or its matrix. Therefore, the analysis of cell spreading and F-actin arrangement helps identify the kind of response induced in each context and subsequently the manipulation of specific physical cues to deliberately induce a stem cell's fate [15].

## 4. Mechanotransduction and Cytoskeletal Rearrangement during Stem Cell Differentiation

Considering that actin filaments are assembled by noncovalent interactions, this provides a higher potential of exchange between the monomeric and the filamentous states. In motile cells, actin dynamics are activated by extracellular stimuli, which in turn trigger intracellular signaling, including Rho-family small GTPases, focal adhesion kinases, cofilin, LIM kinases, capping proteins, polymerization complexes, and other actin binding proteins (ABPs) [18, 19]. Generally, the shift between F-actin and G-actin elicits the acquisition of specific structures and in turn, the change of shape. For example, from spherical to spread shape (or in the opposite direction), the ratio between F-actin and G-actin changes, while in spreading, F-actin helps to generate contact adhesions and stress fibers, as well as membrane protrusions. In both “steady-state” or stimulated conditions, the turnover between filamentous and monomeric actin is continuous [20–22]. G-actin is bound to profilin or thymosin beta 4 (Tβ4). These proteins maintain the stock of G-actin and, along with other ABPs, prevent spontaneous actin polymerization. Profilin can release the G-actin easier than Tβ4 when the polymerization is required [18].

Tβ4 plays a role in human bone marrow-derived MSC differentiation. Tβ4 is known to suppress osteogenic differentiation by sequestering G-actin and preventing its polymerization [23]. It has shown a biophysical effect using exogenous Tβ4, without altering gene expression, measured by monitoring Runt-related transcription factor 2 (Runx2) and peroxisome proliferator-activated receptor gamma (PPARγ) genes during early osteogenic differentiation. Despite a lack of change in gene expression, one cannot rule out the possibility of alteration in other genes not included in this study, since alteration in the actin cytoskeleton may modify gene expression via mechanotransduction. On the other hand, this study has shown that in these conditions, the adipogenic differentiation was promoted, but chondrogenic differentiation was not altered, pointing out the difference in requirements and importance of F-actin during these processes [23].

The cytoskeleton is linked to the extracellular matrix (ECM) by adhesion molecules like integrins, selectins, laminin receptors, and syndecans, and it participates during homing inside an organism or in culture conditions. This contact adhesion also generates a direct link from ECM to the nucleus and confers mechanical properties in a dynamic fashion. In this context, integrins play a crucial role during stem cell maintenance and differentiation. ECM interacts with integrins, and this activates outside-in signaling, while intracellular signaling also induces the conformational change of integrins, as well as modulating its avidity by clustering [24].

**Figure 2:**
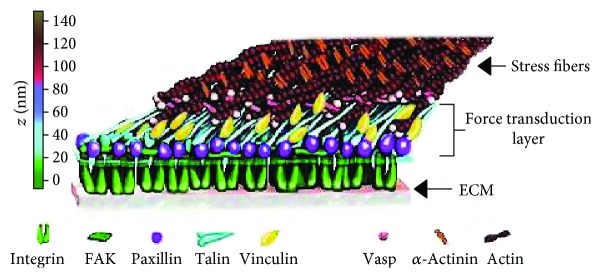
Molecular structure of contact adhesion. Different proteins are involved and are necessary for focal adhesion, like focal adhesion kinase (FAK), talin, vinculin, and paxilin, linking receptors with the cytoskeleton. Outside the membrane, adhesion receptors, like integrins and selectines link the membrane with the substrate (modified from P. Kanchanawong, copyright, 2010).

Integrins may mediate focal adhesions, considered as mechanosensors that link the substrate to the cytoskeleton. From outside of the cell, these structures are integrin clusters, but in the cytoplasm many molecules are recruited to generate a complex structure (Figure 2), with different proteins like focal adhesion kinase (FAK), talin, vinculin, and paxilin, which link receptors with the cytoskeleton [25, 26].

Because stem cells require the activity of integrins, it is important to understand how focal adhesions are regulated and how they can be modulated by biomaterials, inducing more or less adhesion and then altering the differentiation potential of stem cells.

For example, focal adhesion size and intracellular tension induced by ECM proteins regulate stem cell differentiation, as well as self-renewal [27]. Initial contacts and early adhesion generate tension that plays a crucial role in the internalization of signals, including activation of molecules like FAK and mitogen-activated protein kinases (MAPKs).

Myosins are molecular motors that provide mechanical properties and contribute to the tension generated in response to a substrate (adhesion). Myosin II, along with F-actin, generate the actomyosin complex that is largely responsible for establishing the mechanical properties of a cell. Myosin II in smooth muscle and nonmuscle cells is regulated by phosphorylation of myosin light chains (MLC) by myosin light chain kinase [28]. MLC phosphorylation enables the interaction of myosin II with actin filaments and tension generation. Myosin II is also regulated downstream of the Rho family of small GTPases.

The Rho family of GTPases (guanosine triphosphatases) are small molecules involved in regulating actin polymerization. These molecules orchestrate coordinated cytoskeletal reorganizations by generating different structures, like filopodium, lamellipodium, or uropod, depending on the type of GTPase activated, that is, Cdc42, Rac, or RhoA, respectively [29]. In HSCs, the GTPase family also takes part during self-renewal, proliferation, apoptosis, migration, and adhesion. Rac1 and Cdc42 mediate proliferation; Rac2 and Cdc42 mediate apoptosis; and Cdc42, Rac1, and RhoA mediate self-renewal [30]. However, we have to consider that GTPase functions are more complex. In general, these molecules have an activation/inactivation cycle mediated by the exchange of GTP/GDP, respectively. GEFs and GAPs regulate Rho GTPase activity in a stimulus-dependent fashion [31].

As shown in studies with MSCs, RhoA, ROCKII, and the tension that might be generated are important in cell fate, since high activity of RhoA is associated with osteogenic differentiation, while adipogenic differentiation requires a minor activity of the GTPase [32]. In this study they use C3H10T1/2 progenitor cells as a model of primary bone marrow- (BM-) MSCs with a stable population. Expression of Runx2, PPARγ, and Sox9 were analyzed to determine regulation of osteogenic, adipogenic, and chondrogenic differentiation, respectively. By oscillatory fluid flow, the possibility to activate RhoA and then to modulate cellular fate have been measured. This model has shown that RhoA activated by flow may upregulate the expression of Runx2 [32].

Specifically, RhoA/ROCK is involved in the tension induced by fibrous substrates. In this context, RhoA/ROCK signaling generates myosin contractility and this results in increased osteogenic differentiation. In addition, the substrate influences focal adhesion (FA) formation and maturation [33]. Furthermore, by modulating the activity of RhoA it is possible to change the lineage commitment. For instance, the expression of the dominant-negative of RhoA leads hMSCs to differentiate into adipocytes, while the constitutively active RhoA results in osteogenesis [13].

RhoA effectors, ROCK I/II, regulate the actomyosin complex by inhibiting MLC dephosphorylation. Phosphorylation by ROCK inactivates a myosin phosphatase, retaining myosin II in the phosphorylated or active state [34, 35]. ROCK and downstream molecules lead to the interaction of myosin motor molecules with actin filaments [36]. These motor molecules, in association with actin polymerization, contribute to the FAs' assembly, while along with force generation, FAs become more stable and that is known as FA maturation [26].

LIM domain-containing protein kinase (LIMK) regulates actin dynamics by phosphorylating cofilin at Serine3. Cofilin is a member of the actin depolymerizing factors (ADF)/cofilin family of molecules. It was shown that pharmacological inhibition of LIMK1 increased adipocyte differentiation of BM-hMSCs [37], while the opposite result was obtained after siRNA-mediated knockdown of CFL1 and DSTN in human BM-derived primary MSCs, which resulted in osteoblast differentiation [12]. In the same study, the authors analyzed the effect of Cyto-D and phalloidin in human and murine BM-MSCs, confirming that actin polymerization is essential for osteoblast differentiation.

Cofilin is activated by serine/threonine phosphatases like PP1/PP2A [38], calcineurin PP2B [39], Slingshot (SSH1L, -2L, and -3L) [40, 41], and chronophin [42]. Also, membrane phosphoinositides PIP and PIP2 regulate cofilin activity by sequestration. PP2A also regulates phosphoinositide 3-kinase (PI3K), AKT (PKB), mitogen-activated protein kinase (MEK)/ERK, and GSK-3b pathways, which participate in proliferation and apoptosis. In hESCs, PP2A promotes differentiation [43]. This study found incremental activity of PP2A during differentiation of hESCs. Also, inducing the overexpression of this phosphatase or the addition of C2-ceramide (activator of PP2A) promoted differentiation. Hence, by inactivating PP2A with okadaic acid and plurypotency markers, the expression and telomerase activity with normal karyotypes were maintained. This indicated the regulatory role of PP2A in the differentiation/self-renewal of hESCs.

## 5. Mechanobiology: Propagation of Forces through the Cytoskeleton in Linear and Nonlinear Fashions

Since the cell is not a homogenous gel, we have to understand how the forces are distributed and generated by spreading and adhesion. It is necessary to review Newton's Second Law for a better comprehension of this topic. A force is an interaction between two objects, such as stem cells and biomaterials, which may cause acceleration of mass, like gravity, as well as compression (push) or tension (pull) that changes its momentum.

Cells are not static, homogenous entities; on the contrary they are highly dynamic and heterogeneous gels. Analyzing cellular response to a biomaterial is possible, while fixing a point in time as well as a single subcellular compartment. This utilizes Young's modulus, or tensile elasticity, helping measure the extension of cells as a result of biomaterial interaction. In other words, how much a material will deform in response to a stress placed on it can be measured. Materials with a higher Young's modulus are stiffer and do not deform easily. Before differentiation, hMSCs have a Young's modulus value of 3.2 kPa [44].

The disruption of actin filaments causes a decrease in the average elastic modulus of the cellular membrane. Using atomic force microscopy (AFM), Titushkin and Cho [45] reported that osteoblasts have a Young's modulus value of 1.7 kPa. This study also found that hMSCs exposed to an osteogenic medium for 10 days exhibited a significant decrease in the elastic modulus from 3.2 kPa to 2 kPa. Moreover, adipocytes have a lower stiffness than hMSCs and osteoblasts [46]. Darling et al. reported an elastic modulus of 0.61 kPa for adipocytes; this relatively low modulus implies that adipocytes do not have a denser cytoskeleton network in comparison to hMSCs and osteoblasts. The same author reported that chondrocytes have a stiffness of 1.2 kPa, which is a value between that of MSCs and adipocytes (3.2 kPa and 0.61 kPa, respectively) [46].

Tension or compression might be caused by biomaterials or any substrate in which cells will be seeded. As described by Ingber, tensegrity or tensional integrity is the mechanical stability produced by the cellular network and its control of shape and structure [47]. In this regard, the cytoskeletal filament is a network in an isomeric tension state, but it is resilient with a high capability to respond to external forces generating mechanical stress.

**Figure 3:**
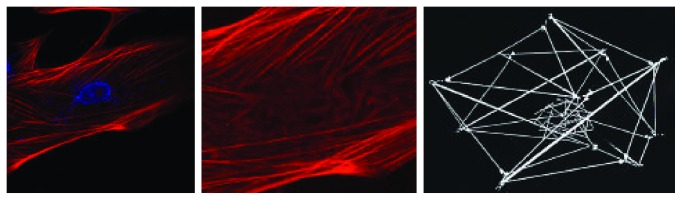
Actin networks of MSCs visualized by a confocal microscope. hMSCs transfected with β-actin-RFP (red) and stained with DAPI (blue) (by Dr. Ambriz, 2016). Actin networks resemble the same pattern from Ingber's model [47] in which tensed elastic strings and straws that are interconnected can predict the actomyosin complex behaviour. Actin filament orientation changes depending on its distance to the nucleus.

Models of tensed elastic strings and interconnected straws can predict actomyosin complex behavior, and the forms created with these models resemble structures observed in living cells [47]. According to Ingber's model, actin networks of hMSCs visualized with a confocal microscope (Figure 3) mirrored the model's pattern, changing the orientation of actin filaments, depending on its localization, whether cortical or closer to the nucleus.

Applied stress by Newtonian fluids can be measured as viscosity. Biopolymers have mechanical properties, ranging from pure fluid to elastic solid, which are considered viscoelastic [48]. Cells also respond to the intrinsic properties of biomaterials by restructuring F-actin networks, complicating the measurement of force distribution along the cytoplasm or cellular membrane [49]. Moreover, ABPs provide a dynamic way to rearrange actin filaments, linking or severing F-actin, as well as by conferring greater elasticity to the filament. Cofilin association to F-actin changes the filament twist in ∼4 to 5° per subunit [50]. Therefore, the cytoskeleton network displays a nonlinear elastic response [46], since stiffness or softening are regulated by F-actin and myosin II, not just actin. This was first suggested by Eliot Elson in the 1990s. Subsequently, Cai et al. showed that myosin light chain kinase plays a crucial role in regulating cell stiffness by phosphorylating myosin II [51]. These cellular characteristics and properties have to be included in the design of biomaterials and scaffolds, as well as stem cell therapies, since they can transduce signals provided by nanotopography and stiffness of biomaterials.

## 6. Nanotopography of Biomaterials and the Role of the Cytoskeleton during Stem Cell Interaction

Actin and tubulin, in contrast to vimentin, are cytoskeletal molecules generating, in a highly dynamic fashion, specialized structures in just a few seconds after a signal is initiated. This property provides a cell the capability of adapting if the microenvironment changes, or if it is required in other processes, like cell migration.

**Figure 4:**
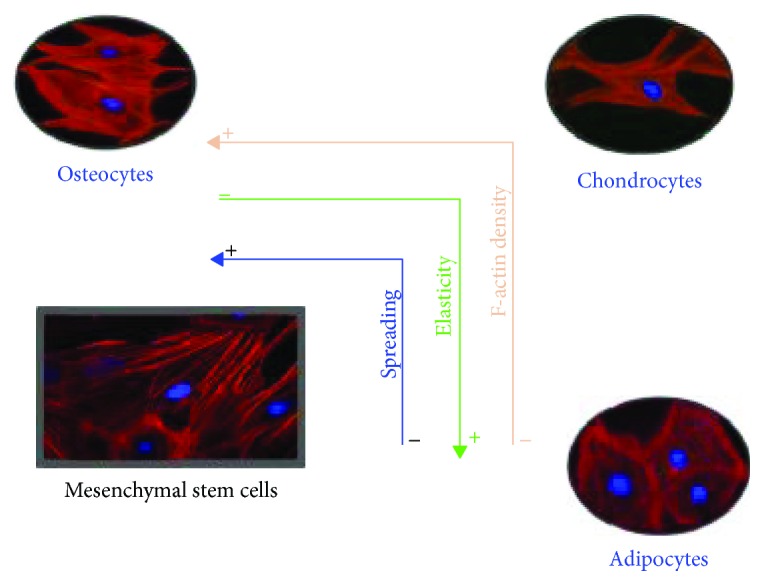
Actin cytoskeleton in stem cell differentiation. Cellular spreading (blue line), elasticity (green line), and F-actin density (orange line) are some of the properties that change during stem cell differentiation and can predict the cell's fate. Substrates with different mechanical characteristics influence the rearrangement of the F-actin cytoskeleton in the differentiation process modulating cellular spreading, elasticity, and F-actin. In the case of adipocytes, they have less F-actin and spreading but are more elastic compared to chondrocytes and osteocytes. On the other hand, osteocytes have the opposite context than adipocytes. They are less elastic but with higher F-actin and spreading. Chondrocytes display intermediate properties of adipocytes and osteocytes considering these three properties (by Dr. Ambriz, 2018).

Stem cells share an exceptional plasticity and adaptation to microenvironmental conditions. According to the plasticity potential, cells respond and adhere easily, acquiring a spherical or spread shape. Stem cells are unspecialized with a remarkable potential to renew themselves, as well as the potential to differentiate into mature specialized tissue; both functions are maintained in balance. With *in vitro* models, these properties can be manipulated by employing different strategies, like cocktails of growth factors. Another approach is to use scaffolds, like mechanical cues [16, 17], which can be manipulated to generate specific changes in cellular shape, spreading, rearrangement of F-actin, stress fiber formation, and inducing adhesion patterns and differentiation [52, 53] (Figure 4).

In the human body, stem cells interact with ECM, which vary in composition depending on the site. ECM is mainly composed of collagen, fibronectin, and laminin [54], as well as proteins like glycosaminoglycans and proteoglycans. Niche compositions rely on the abundance of these molecules and contribute to the differentiation process in which stem cells generate specific adhesion arrangements, maintaining or modifying its cellular shape and migration capability to other sites.

When stem cells are maintained in culture, it is crucial to avoid any possible stimulus that can trigger the differentiation process. For example, fibronectin binds to integrins like α5β1, α4β1, and αvβ3. This interaction results in an intracellular actin reorganization and induces a specific change of shape and the activation of intracellular signaling pathways, which results in chondrocyte differentiation. On the other hand, in vitro self-renewal of ES cells has been shown to be dependent on the interaction with type I or type IV collagen substrates [55].

Induction of morphological changes of hMSCs have been studied by the generation of specific micropatterns [25, 56] causing topography-mediated differentiation [56]. ECM proteins have been used to “print” patterns, lines or dots, with different spacing between each one, resulting in the induction of tension or compression, as the cells attempt to generate different distributions of FAs.

To illustrate the influence of nanotopography, consider nanoislands of fibronectin. With spacing below 60 nm, it is possible to generate FAs, while longer spacing results in impairment of FA formation and cellular spreading. Moreover, the organization of patterns seems to be significant since disordered nanopatterns do not cause cellular spreading. Furthermore, FA maturation and cell adhesion strength were constant at 60 nm of spacing, in comparison to 70 and 120 nm of micropatterned substrates [25]. These results are highly significant if it is considered that biomaterials, with specific micro- or nanopatterned designs can modulate the maturation of FAs, which in turn has consequences in stem cell proliferation, self-renewal, and differentiation, as described above.

Adhesion structures are required sites for binding to the substrate. These molecular structures function as links between the substrate and the actin cytoskeleton. Furthermore, motor molecules like myosins, FAKs activated by integrins, and the activation of the molecular machinery responsible for actin polymerization generate mature or immature contact adhesions. These contacts in turn modify the affinity and valency of integrins and are the main features during the adaptation of stem cells to a surface. The molecular machinery involved and adhesion structures' organization that was generated rely on the mechanical properties of the substrate.

For example, the stiffness of the substrate influences adhesion structuring as well as stress fibers, establishing the actomyosin complex. If the substrate is soft, the shape of the cell will be more rounded with a low density of stress fibers. If substrate stiffness is increased, the density of stress fibers and the spreading of the cell will also increase [54–56]. The stiffness of the substrate can be manipulated, allowing the design of specific conditions and the induction of stem cell differentiation.

Other techniques exist, measuring the physical forces and mechanical properties of cells, making possible the analysis of conformational changes by AFM and Förster resonance energy transfer (FRET). Single cell analysis of mechanobiology is also possible by microelectromechanical systems (MEMS), AFM, optical stretchers, and micropipette aspirations [56]. These techniques create standards required to control stem cell differentiation.

Mechanical forces produced during the interaction between substrate or biomaterials and stem cells cause mechanical stimulus, and this may be translated in intracellular signaling, both chemical and biophysical [57, 58]. In this context, the actin cytoskeleton plays an active role in the interpretation of the microenvironmental conditions that drive and link directly to the cytoplasm and nucleus, having the consequence of molecular activation and/or gene transcription [59–62].

## 7. Cancer Stem Cells: Targeting Their Biomechanics

The complexity within stem cell niches elicits the interaction of CSCs with other cells. All of them could be involved in some kind of regulation but these aspects need to be defined specifically, as they are all related with those molecules involved in biophysical interactions of CSCs. Inside of cancer cell niches, several types of cells exist, such as tumor, stromal, and vascular cells where as part of them, CSCs are maintained in a quiescent stage until adequate conditions, including cellular and molecular events, induce them to proliferate, invade, and metastasize [63].

The expression “awakening the beast” has been used to describe the activation of CSCs following chemotherapeutic treatment [64]. CSCs have clear properties of self-renewal, clonal tumor initiation capacity, clonal long-term repopulation potential, transitions from a nonstem cell state to stem cell state, evasion of cell death, metastasis, and dormancy for long periods of time [65]. CSCs can also be activated by direct or indirect interactions with different cell types present inside of niches [65, 66] and by biophysical interactions within cancer cell niches surrounding ECM or ECM molecules [67, 68]. In those niches, the local cells produce factors capable of stimulating CSCs, inducing angiogenesis, and recruiting immune and other stromal cells that secrete additional factors while promoting tumor cell metastasis and invasion. CSCs can also produce exosomes, which facilitate ingress of RNA molecules that facilitate the ingress of multidrug resistance in tumor cells [65].

In tumor niches or tissues, there is a production and concentration of several molecules that could activate CSCs. Biochemical and biophysical signals come from growth factors, cytokines, and matrix-remodeling proteins [63]. Potentially, all of them are capable of activating and/or inducing growth and differentiation of quiescent CSCs that develop into more aggressive stages. Part of those molecules are noncellular components derived from degradation of ECM due to the action of matrix metalloproteinases coming from activated cells inside of niches; the importance of the produced ECM is due to its constitution remaining as a physical barrier, as well as the integrity of CSCs blocking any possible harmful condition such as the action of chemotherapeutic agents found in solid tumors. Therefore, the release of cytokines, growth factors, and other molecules enable the degradation of the ECM because of the action of metalloproteinases which are factors facilitating angiogenesis, tumor cell metastasis, and invasion associated with therapeutic resistance. Cross talk between CSCs and their niches have the basis of activation of these types of cells [65].

It is clear that those factors present in the microenvironment of cells have an important influence on different stem cell phenotypes. Such factors are composed of materials surrounding the cells that can compete with biochemical supplements. They influence or induce the activation and/or the differentiation of stem cells by inducing or activating signaling pathways through mechanotransduction because of its mechanosensing [68]. Therefore, those conditions involving cancer therapy, where there is a manipulation of the components of the microenvironment of cancer niches or tissues, could produce an effective strategy for cancer treatment or the induction of resistance to cancer therapy and the prevention or the maintaining of malignancy and metastasis of CSCs [65].

Materials in cellular environments are capable of being inductors/activators of stem cells which is a crucial consideration for CSC niches. If these molecules are part of the surrounding niches, their surfaces could become inductors of CSC activation that can be involved in mechanotransduction and mechanosensing events (as those described in the present revision for stem cells). These conditions could trigger growth, expansion, and drug resistance as shown by these types of cancer cells. It is mandatory to consider whether their engineering and utilization could be the reason why some quiescent CSCs become capable of expanding and developing resistance to anticancer drugs [69]. There are several properties of synthetic materials which can induce changes in cellular activity, and these include stiffness, molecular flexibility, nanotopography, cell adhesiveness, binding affinity, chemical functionality, degradability, and/or degradation by-products. Materials could produce specific stem cell behavior which need to be always considered during the design and use of materials [68], and CSC biology and niche factors have to be involved during the use of materials for regenerative medicine purposes.

The significance of cell fate and mechanotransduction has been discussed above, but to understand how they are involved in CSCs' progression to metastasis requires describing the signaling pathways activated when a mechanical disruption occurs and cell-cell contact is lost. There is a tensional homeostasis within cells when there is a disruption, and it may play a role in oncogenic transformation. Considering that stiffness is measured by mechanosensors in cancer stem cells and solid tumors have high mechanical stress, this may impede drug delivery driving tumor progression. Biomechanic forces can drive tumor aggression in the case of a mesenchymal-like switch, developing tumor-initiating or stem-like cell properties, as well as elevated tissue mechanics promoting aggression. These events open the possibility of manipulating mechanical properties of CSCs to break drug resistance by its stem cell phenotype [70].

## 8. Signaling Molecules Involved in Biomechanics and Drug Resistance in Cancer Stem Cells

In most solid tumors, recent studies show the relevance of the oncogenes Yes-associated protein (YAP) and transcriptional coactivator with PDZ-binding motif (TAZ) transcriptional regulators. YAP/TAZ activation provides not only stem cell properties in cells, but also chemoresistance. Considering these molecules as mechanosensors, it is highly relevant to understand its function in normal or pathological conditions and how its activation potentially is a key factor for cancer treatment and designing anticancer drugs [71, 72].

YAP/TAZ proteins are mechanosensors and mechanotransducers that respond to physical stimulus in which the actin cytoskeleton is involved, such as the rigidity of extracellular matrix, cell geometry, cell density, and cell polarity [73]. For this purpose, it is important to maintain that molecules in ECM are polymers such as collagen or fibrin, which also self-ensemble into gels. For example, increasing the amount of collagen in a tissue enhances its stiffness. Moreover, cytoskeletal stress causes protein stabilisation to maintain cell integrity, and one good example is the nuclear structure protein lamin A, whose protein and transcript levels increase with collagen-I and tissue stiffness. Then, mechanogenomic processes are pivotal inducers in mutations and cause cancer related to tissue stiffness [74]. Approaches to reduce tumor aggression include reducing or inhibiting TGFβ, LOXL2, or collagen deposition and cross-linking [70].

YAP/TAZ have been shown to play positive as well as negative roles in the Wnt signaling pathway. This pathway is important to measure cell-cell contact, among other functions. YAP/TAZ are integral components of the β-catenin destruction complex, which they may translocate to the nucleus after Wnt pathway activation [75]. Other hypotheses support the notion that ECM stiffness is the extracellular activator of YAP/TAZ downstream G protein coupled receptor (GPCR)/lipid rafts/Rho/ROCK signaling pathway, leading to CSC survival [76, 77].

On the other hand, integrin and FAK activation result in focal adhesion formation, which in turn activates Rho-GTPases and elicits the formation of stress fibers. This leads to the activation of other kinases such as LATS1/2 and inhibition of YAP/TAZ transcription factors causing a negative effect in CSCs [76, 77]. In this context, actin polymerization is required in focal adhesions. After cellular spreading, myosin II increases tension on the actin network while depolymerization of F-actin decreases the tension, and this maintains the tension in equilibrium.

Mechanical properties of CSCs will be essential in the design of personalised medicine in order to develop more efficient treatments. Then, it is necessary to consider the disruption of tumor microenvironment barriers, such as stromal cells, vasculature, and collagen cross-linking, which restrict drug entry and then affect the chemotherapeutic efficiency. If there are well-designed nanomaterials [78] and nanoparticles [79], such as nanocarriers used to reach the target cell, it will improve drug delivery and provide access for drugs to cancer cells, including CSCs. Also, biomaterials which may modulate and reduce the chances of wrong activation of the CSCs should be designed.

## 9. Conclusions and Perspectives

Mechanotransduction helps comprehend the mechanism by which the nanotopography of biomaterials can direct the differentiation of stem cells. Intracellular tension generated by the adhesion to a biomaterial is transduced like a chemical signal with the possibility of gene expression. In this manner, interactions with physical cues provided by nanotopography, nanopatterns, stiffness of the substrate, and other physical properties of biomaterials, can modulate stem cell fate and must be considered in the study of these cells and the use of stem cells in regenerative medicine and cellular therapy.

Understanding cytoskeleton arrangement and its implication during stem cell interactions with biomaterials, as well as the importance of nanotopography, creates new aspects of integration with other fields of knowledge, in order to improve cell-based therapies and regenerative medicine.

Along with the considerations of the present work, clearly it is mandatory to have new ways of focusing the design and engineering of new biomaterials with intentions of using it for therapeutic purposes. Not taking into account these considerations could be a determinant for inducing abnormal activation or differentiation of stem cells.

It is a big risk to design and build a biomaterial without evaluating their physical aspects, such as the stiffness, porosity, nanotopography, chemical composition, and interaction with other types of surfaces. Moreover, the aberrant growth of cells or the wrong differentiation of the stem cells can be triggered by alterations in their physical microenvironment. As indicated, under these considerations, activated cells could be redirected to produce pathological situations or in the case of CSCs to develop resistance to anticancer drugs due to epigenetic changes. It is important to carry out the characterization of the new biomaterials, in order to establish if they are adequate for the terms of any potential use in regenerative medicine and, more importantly, if they do not have the capability to “awake the beast.”

In conclusion, the mechanobiology of stem cell differentiation and cytoskeletal dynamics provide the knowledge to develop cellular therapies with higher efficiency, as well as the possibility of understanding its involvement in pathological conditions and its effect on cellular biology.
